# Modelling African horse sickness emergence and transmission in the South African control area using a deterministic metapopulation approach

**DOI:** 10.1371/journal.pcbi.1011448

**Published:** 2023-09-06

**Authors:** Joanna N. de Klerk, Erin E. Gorsich, John D. Grewar, Benjamin D. Atkins, Warren S. D. Tennant, Karien Labuschagne, Michael J. Tildesley

**Affiliations:** 1 The Zeeman Institute for Systems Biology and Infectious Disease Epidemiology Research, School of Life Sciences and Mathematics Institute, University of Warwick, Coventry, United Kingdom; 2 South African Equine Health and Protocols NPC, Baker Square, Paardevlei, Cape Town, South Africa; 3 Epidemiology, Parasites and Vectors, Agricultural Research Council, Onderstepoort Veterinary Research, Onderstepoort, South Africa; Fundação Getúlio Vargas: Fundacao Getulio Vargas, BRAZIL

## Abstract

African horse sickness is an equine orbivirus transmitted by *Culicoides* Latreille biting midges. In the last 80 years, it has caused several devastating outbreaks in the equine population in Europe, the Far and Middle East, North Africa, South-East Asia, and sub-Saharan Africa. The disease is endemic in South Africa; however, a unique control area has been set up in the Western Cape where increased surveillance and control measures have been put in place. A deterministic metapopulation model was developed to explore if an outbreak might occur, and how it might develop, if a latently infected horse was to be imported into the control area, by varying the geographical location and months of import. To do this, a previously published ordinary differential equation model was developed with a metapopulation approach and included a vaccinated horse population. Outbreak length, time to peak infection, number of infected horses at the peak, number of horses overall affected (recovered or dead), re-emergence, and R_v_ (the basic reproduction number in the presence of vaccination) were recorded and displayed using GIS mapping. The model predictions were compared to previous outbreak data to ensure validity. The warmer months (November to March) had longer outbreaks than the colder months (May to September), took more time to reach the peak, and had a greater total outbreak size with more horses infected at the peak. R_v_ appeared to be a poor predictor of outbreak dynamics for this simulation. A sensitivity analysis indicated that control measures such as vaccination and vector control are potentially effective to manage the spread of an outbreak, and shortening the vaccination window to July to September may reduce the risk of vaccine-associated outbreaks.

## Author summary

African Horse Sickness is a devastating global equine disease which results in high numbers of deaths. In South Africa it is considered endemic, and this poses implications for trade and exportation of horses out of the country. As a result, South Africa has developed a “control area” where there are increased surveillance and control strategies to keep it free from African horse sickness.

Our research explores where and how an outbreak may occur in the “control area” if a horse harbouring the virus were to be imported into the region. We developed a mathematical simulation which considered the differences in horse population numbers, midge population numbers, environmental temperature, and vaccination status of horses in different areas across the “control area”. This helped to determine how many horses would be affected at the end of the outbreak as well as at the peak, how long the outbreak would occur, and the reproduction number (R_v_), and how this varied across the study area. Our research uncovers insights into the African horse sickness outbreak dynamics of the “control area” and helps provide information to further develop control strategies.

## Introduction

African horse sickness (AHS) virus is an equine orbivirus spread by *Culicoides* Latreille biting midges. It is endemic in Sub-Saharan Africa and circulates among a large equine population of horses, mules, donkeys, and zebras. Infected equids can be affected by high morbidity and mortality rates [1] and the disease can manifest in three forms: the cardiac form (dikkop), respiratory form (dunkop), and mixed form, each of which have differences in incubation time and mortality rates. The respiratory form is the most common, characterised by a fever, coughing, breathing difficulties, inflamed conjunctiva, and oedematous supraorbital fossae. The incubation period is typically 3–5 days. Recovery is rare and death occurs within a week. The cardiac form is often subacute for a longer period than the respiratory form with an incubation period of 1–2 weeks. Visual symptoms of the cardiac form include a fever, combined with facial, neck, and thoracic swellings. The associated mortality rate is around 50% and if death occurs, it happens around one week after the onset of symptoms [2]. Mixed forms can also develop with a mortality rate of around 80% [2]. In addition to the severe clinical manifestations of African horse sickness (AHS), subclinical or mild infections are also common, particularly in other equine species [3] and partially immune horses with lapsing vaccinations [4]. Levels of viraemia in subclinically infected equids may be sufficient to infect susceptible midges, however the role of subclinical infections in the epidemiology of African horse sickness requires further investigation [5]. Nonetheless, subclinical infections have been evident in previous outbreaks, sometimes in high numbers, such as the 2016 Paarl outbreak with 64% identified cases being subclinical [6].

Outbreaks of AHS also impact the safe movement of horses nationally and internationally. For example, between January 1997 and December 2018, the trade of live equids between South Africa and the EU was only viable around 44% of the time due to circulating AHS [7]. Each outbreak resulted in a two-year post-outbreak suspension in trade, as legislated by the European Commission [7]. Other major global AHS outbreaks impacting the horse industry have occurred in southern Europe in 1966 and 1987–1990, the Far and Middle East in 1944 and 1959–1963, frequently North Africa, due to spread of the disease up the Nile valley and African West coast [8], and most recently Thailand and Malaysia in 2020 [9]. Nevertheless, restriction of trade and movement of equids is only one of the many control measures used to combat AHS outbreaks. Active surveillance, equine vaccination, chemical vector control, physical vector barriers, alterations in horse management, such as stabling and avoiding riding at dawn and dusk, and environmental hygiene maintenance to reduce midge breeding grounds, are frequently implemented to reduce the ability of the disease to spread.

Modelling to guide the control of AHS is limited by the complexity of the disease and its response to vaccination. African horse sickness virus has nine serotypes. A live-attenuated polyvalent vaccination is available, which provides protection across all nine serotypes; however, it can take up to six years (or eight vaccination courses) before effective immunity develops in an animal [10]. This, combined with individual variation between horses in terms of age and co-morbidities, and the presence of horses with lapsed vaccinations, frequently results in a large disparity in AHS immunity which can complicate the modelling process. In addition to the complexities presented within the horse population, variations in environmental factors, such as temperature, vegetation, rainfall, and altitude, influence *Culicoides spp*. population dynamics. Midge biting rate, mortality rate, and viral incubation time are all temperature dependent and therefore variations in the environment need to be considered when developing an AHS model [11].

Lord *et al*. (1996) were the first to model the disease dynamics to determine what an AHS outbreak in Spain might look like after the introduction of the virus [12]. However, at this time there was limited computational power and minimal data to base the model on [4]. In the years that followed, the European bluetongue epidemic resulted in a considerable increase in knowledge, as more information was collected on orbiviral transmission and how midge populations vary in time and space. With the gain of this knowledge about infection biology, Backer and Nodelijk (2011) considerably adapted the AHS model by representing midge-to-horse transmission in the Netherlands if an infected horse were to be imported [4]. This vector-host model was then used as a basis for Porphyre and Grewar’s model exploring AHS transmission in zebra populations in South Africa [13], and the parameters have recently been further updated following a systematic review [14]. Nevertheless, at the time of this publication, there had not yet been a model which has incorporated the complexities of an equine population where a proportion is vaccinated, nor addressed the potential for spatial spread of infection via midge dispersal. Given that vaccination seropositivity ranges from 25% to 100% depending on the number of doses [10] and midges can fly up to 3km per day [15], they both likely play an important role in transmission.

This model focusses on AHS in South Africa, as the country presents a unique approach to managing the disease to establish an AHS-free area despite AHS being endemic in the country. In 1997, the ‘AHS control area’ was established in the Western Cape Province where safe exportation of horses to AHS-free countries can occur. It is separated into three zones: the free zone (Cape Town and surrounding areas), the surveillance zone (the remainder of the City of Cape Town municipality, Saldanha Bay, Swartland, Drakenstein, and Stellenbosch), and the protection zone (Bergrivier, Cederberg, Witzenberg, Breede Valley, Langeberg, Theewaterskloof, Overstrand, Cape Agulhas, and Swellendam) as was mapped out by the Department of Agriculture, Forestry and Fishing [16] (now known as the Department of Agriculture, Land Reform and Rural Development–DALRRD). For the remainder of the country, AHS is considered endemic. Within the free and surveillance zones, vaccination can only occur with annual permission from the state vet, as sporadic outbreaks have occurred due to virulent reversions of the AHS vaccine [17]. However, vaccination permission is often granted to facilitate movement, competition, and insurance requirements. In these zones, it is estimated that 40–50% of horses are vaccinated, based on vaccination permission reports from the last four years [18]. In contrast, in the surrounding protection zone, all domestic equids must be vaccinated during the winter (1^st^ June to 31^st^ October) when midge abundance is lower. In the rest of the country, AHS vaccination is required but can be undertaken during anytime of the year. Unfortunately, compliance is poor and there is no data available about the true level of vaccination in the endemic area.

In the AHS control area, there have been eight AHS outbreaks between 1999 and 2021, of which the 1999 Stellenbosch outbreak in the surveillance zone and the 2006 Robertson outbreak in the protection zone were thought to be due to illegal importation of infected horses into the area [19]. The source of the Cederberg protection zone outbreak in 2021 was unknown, although wind dispersal of midges was concluded to be a likely scenario [20]. The remaining outbreaks were either due to reassortment or reversion to virulence of the vaccine. All recorded outbreaks in this area were initiated between the start of February and early April and lasted between 32 and 83 days and there has not yet been a case of AHS overwintering that is known about. While the incidence of an outbreak occurring due to importation is very small, it still presents a risk with severe consequences for equine movement and trade.

In this paper, we extended a mathematical model previously developed by Backer *et al*. (2011) [4] by representing vaccination, vaccine efficacy and midge population dynamics in our study area, which is further discussed in the methodology section. Using our updated model, we explored the AHS risk within the South African control area and aimed to provide valuable insights regarding the spatiotemporal variability in outbreak dynamics should a latently infected horse be accidentally imported into the control area, as well as how control measures could impact an outbreak if it were to occur.

## Results

### Simulation example

**Fig 1:**
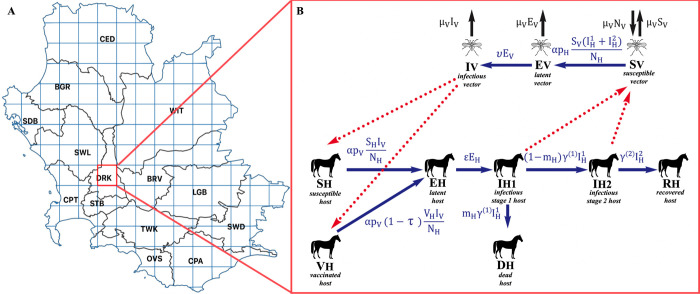
Set up for the epidemiological model in the African horse sickness (AHS) control area. (A) Map of the AHS control area overlayed with a grid made up of 153 cells in which the model was run. The highlighted cell is the example simulation cell. Municipalities within the study area were CPT = City of Cape Town, SWL = Swartland, DRK = Drakenstein, STB = Stellenbosch, OVS = Overstrand, TWK = Theewaterskloof, BRV = Breede Valley, SDB = Saldanha Bay Bay, BGR = Bergrivier, CED = Cederberg, WIT = Witzenberg, LGB = Langeberg, CPA = Cape Agulhas, SWD = Swellendam. (B) Schematic diagram showing the transmission pathways of the AHS model in South Africa, and the calculations used in the model. Blue arrows indicate the movement of horse or midge individuals from one compartment to the next, whereas red, dotted lines indicate the transmission of AHSV from host to vector or vector to host. The base layer of the map is publicly available to download from the Municipal Demarcation Board of South Africa (2018) [21].

**Fig 2:**
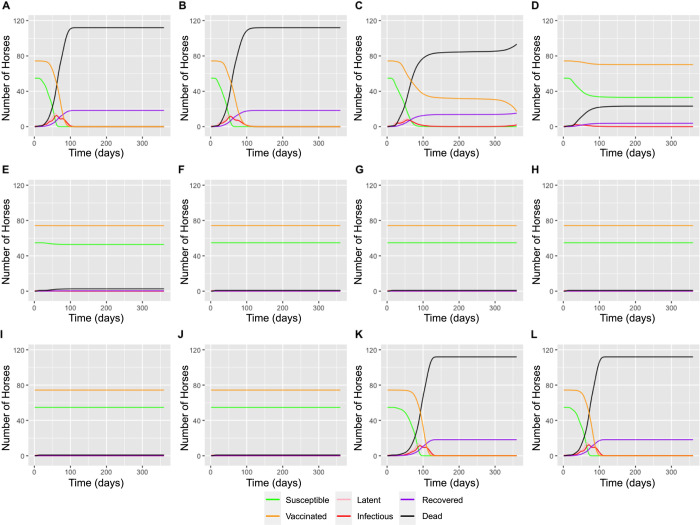
Graphs showing the outputs of the model simulation. Line graphs demonstrating the changes in the horse population when the simulation was run for 360 days varying the start date from January to December (A-L).

**Fig 3:**
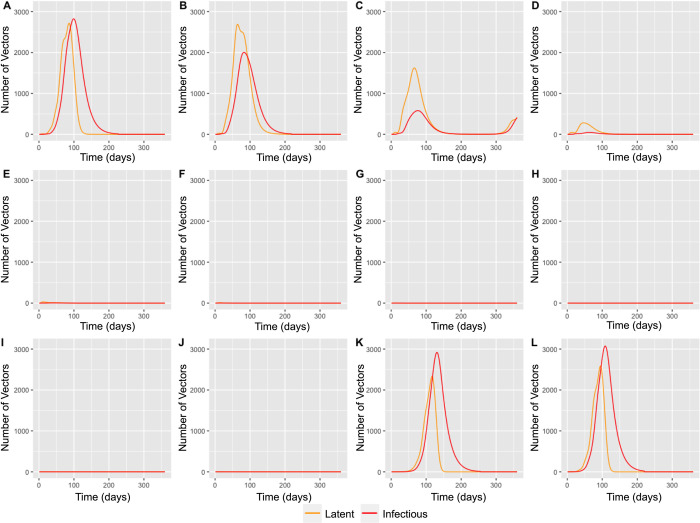
Graphs showing the outputs of the model simulation. Line graphs demonstrating the changes in the midge vector population when the simulation was run for 360 days varying the start date from January to December (A-L).

The simulation was run for an example location (cell ID 84) in the heart of the AHS control area in the municipality of Drakenstein in the surveillance zone (Fig 1A). Here, 50% of the 32 horses were vaccinated with a vaccine protection factor of 95% and neighbouring locations housed 98 more horses with vaccination cover ranging from 50% to 72.5% and vaccine protection factor of 94.5% to 95%. The temperature ranged throughout the year between 11.9°C in July to 22.9°C in January, and the midge count dipped in October, with a count of 388, then peaked in April, with a count of 7802 midges. The sine wave and Periodic Gaussian function fitted to the temperature and midge counts, respectively, fit well (S1A and S1B Fig). Using the parameters detailed in Table 1, the simulation gave rise to the dynamic horse and midge behaviour demonstrated in Figs 2 and 3.

**Table 1:** Parameters used in the model. Parameters were informed by the re-parameterised AHS model developed by Fairbanks (2022) [14], the Netherlands AHS model by Backer and Nodelijk (2011) [4] and the bluetongue model by Szmaragd et. al. (2009) [36].

Parameter	Description	Value
*ε*	Latent period for hosts (days) = 1/*ε*	4.6
*l*	Number of stages for latent host class	5
*γ* ^(1)^	Overall recovery rate from 1^st^ infectious host class	0.26
*n* ^(1)^	Number of stages for 1^st^ infectious host class	11
*γ* ^(2)^	Overall recovery rate from 2^nd^ infectious host class	1.25
*n* ^(2)^	Number of stages for 2^nd^ host class	13
*m*_*H*_	Mortality of hosts	0.86
*T*	Temperature in month of simulation (degrees Celsius)	10.6–25.4
*α*	Blood feeding interval = 1/*α*	1 / (0.015 *T*– 0.125)
*υ*	Extrinsic incubation period (days)	0.0085 *T*– 0.0821
*k*	Number of stages for incubating vector class	10
*μ*_*V*_	Average life span (days)	0.015 exp (0.063 *T*)
*p*_*H*_	Probability of transmission from host to vector	0.52
*p*_*V*_	Probability of transmission from vector to host	0.77
*τ*	Vaccination protection factor	0.52–0.95
Ω	Change in midge population (x)	f’(x)
*κ*	Kernel parameter	0.034
*ρ*	Force of infection between cells (*d* = distance between centroids)	$\rho (d)=\frac{\kappa }{\surd \pi }exp(-\kappa^{2}d^{2})$

**Fig 4:**
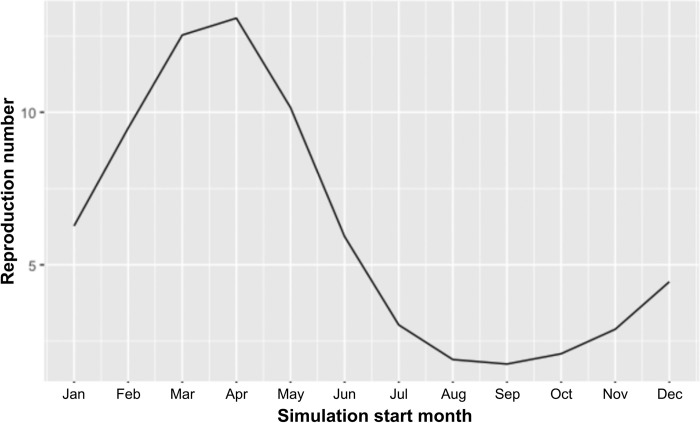
Graph showing the outputs of the model simulation. Time-series graph demonstrating how the reproduction number (R_v_) varied depending on the start month of the simulation.

The simulation was run starting in each month of the year, to explore how the outbreak might vary if a latently infected horse was transported into the cell during each month. Outbreaks did not take off following importation of a latently infected horse in May to October. November through February demonstrated similar outbreak dynamics, with a sharp rise in infected horses, coupled with a sharp decline in susceptible horses, and a similar decline after a slight delay in vaccinated horses. Starting in March, the outbreak had an initial peak before appearing to die out, where it experienced a period over the winter when no further infected horses were noted. Nevertheless, a very small number of latent and infected midges overwintered (Figs 3 and 4), resulting in a second wave of the outbreak as the temperatures increased in the spring, predominantly infecting the vaccinated horses which did not die out in the initial wave. Finally, April experienced a much smaller outbreak with fewer horses and midges becoming infected before the winter months resulted in the outbreak dying out. The starting month with the most infected horses at the peak was January with 12 infected horses out of a horse population size of 129 between the cell and its neighbours. This was different from the month with the greatest infected midge peak, which was December with a peak of 3073 infected midges out of a total of 25,288 between the simulation cells and neighbouring cells. In addition to this, in the months November, December, January, and February all the horse population were affected during the outbreak, whereas the midge population was never fully infected due to the short life span of a midge and the constant birth, and therefore renewal, of midges in the susceptible population. The basic reproduction number in the presence of vaccination (R_v_) varied from 1.75 to 13.08, with the highest value being for outbreaks starting in April and the lowest being for outbreaks starting September (Fig 4).

### Study area spatial epidemiology

**Fig 5:**
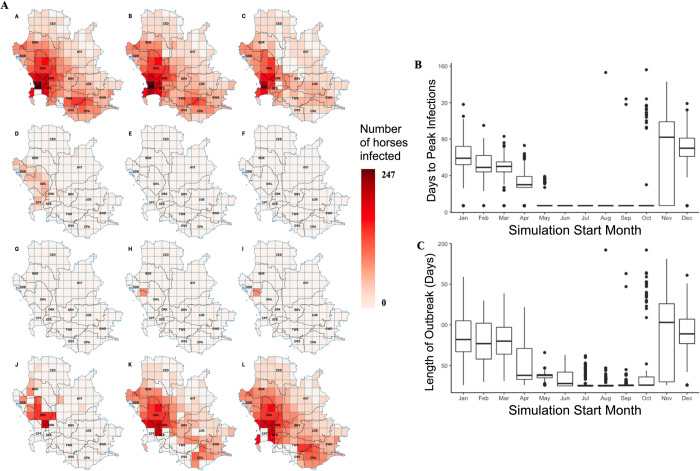
Overview of the outbreak dynamics in the AHS control zone from the model simulation. (A) Spatiotemporal maps showing the number of affected (dead or recovered) horses in the outbreak with varying start months of the simulation from January to December (A-L). (B) Box plots displaying the number of days to the peak infection day depending on the start month of the simulation. (C) Similarly, box plots displaying the length of the outbreak in days depending on the start month of the simulation. The base layer of the map is publicly available to download from the Municipal Demarcation Board of South Africa (2018) [21].

Geographical differences in horse population size, vaccination coverage, vaccination efficacy, midge count, and temperature accounted for the heterogenous results across the study area (Figs 5A and S3A–S3E).

The number of days to the peak of the outbreak (S3A Fig) and the outbreak length (S3B Fig) followed similar trends, where a longer time to reach the peak usually also resulted in a longer outbreak. The warmer months (November to March) had longer outbreaks than the colder months (May to September), took more time to reach the peak, and had a greater total outbreak size (Fig 5A) with more horses infected at the peak (S3C Fig). This was particularly evident in the surveillance zone where the horse population sizes are greater and vaccination cover is less. In these months, outbreaks were more common, with longer, slower outbreaks occurring at the end of the year (early summer), and shorter, faster outbreaks occurring at the start of the year (late summer) (Fig 5B and 5C). During colder months, infection was often limited to only the initial imported horse or were limited to only a few horses, and quickly died out when the infection was not further spread. Nevertheless, the spring (October and November) and autumn (April, May, and June) months displayed unique characteristics where the initial outbreak waves did not appear too severe and seemed to die off in the horse populations, but potentially small numbers of infected midges survived. The spring initiating outbreaks in the surveillance zone were more likely to demonstrate a re-emergence and a second outbreak wave after winter, and the autumn initiating outbreaks in the north of the protection zone experienced second outbreak waves in late summer (S3D Fig).

The reproduction number (R_v_) was under one for large areas of the control area for outbreaks starting between the months of July to October and remained relatively low throughout most of the control area for the remainder of the year (S3E Fig). However, in February, March, and April, which were the months which experienced the hottest temperatures and largest midge population sizes, R_v_ greatly increased, particularly in the east of the protection zone. Geospatial similarities can also be observed, where mountainous areas running from the north to south-east have lower temperatures, and therefore lower R_v_ values, as well as the southern areas with lower midge and horse densities, also experience lower R_v_ values. Nevertheless, the patterns observed in the simulation did not similarly result in more severe or larger outbreak sizes in this area. Therefore, R_v_ appeared to be a poor predictor of outbreak dynamics for this simulation.

**Fig 6:**
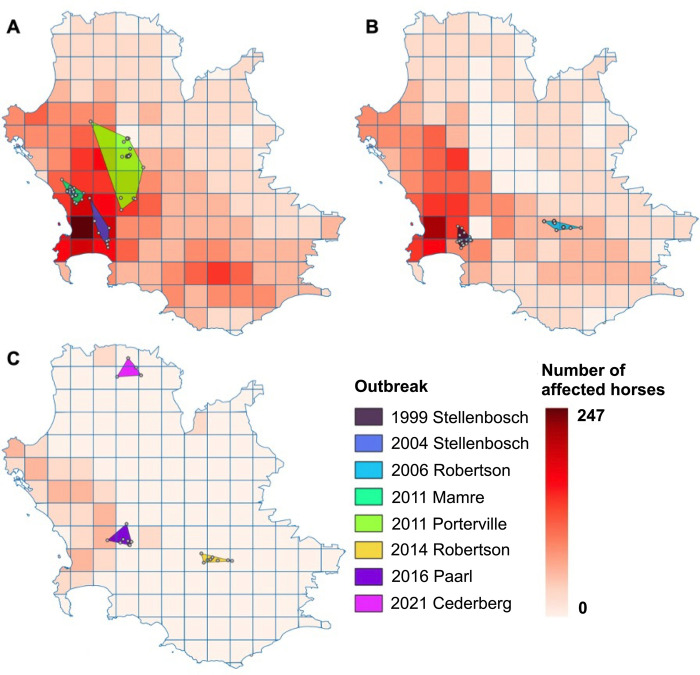
Maps displaying the locations of the eight known AHS outbreaks in the control area, and the number of affected horses in the simulation, based on the months they originated in. Months included February (A), March (B) or April (C). The base layer of the map is publicly available to download from the Municipal Demarcation Board of South Africa (2018) [21].

The simulation successfully identified areas with similar outbreak dynamics where observed outbreaks occurred (Fig 6). Observed outbreaks are described in S1 Text. They occurred from 1999 to 2021 and include two natural imported infections, one natural infection from an unknown cause and five vaccine-related outbreaks due to reversion to virulence or reassortment of the genome of live-attenuated vaccine strains. Outbreak dynamics were not compared to vaccine-related outbreaks because it is unknown whether they share the same transmission parameters and infection biology as natural infections. Also, two known natural infections were in 1999 and 2006, when the case definition and diagnostic techniques were not as advanced as they are today, likely resulting in many cases being unidentified. Consistent data collection would facilitate further model validation.

### Sensitivity analysis

Six parameters were evaluated using a sensitivity analysis by varying the inputs and exploring the impact of variation on the outcomes of the model (S4 Fig).

Varying the vaccine protection factor and the proportion vaccinated results in large changes to the outbreak dynamics in terms of the number of horses infected at the peak, outbreak length, overall size of the outbreak, and R_v_ (S4 Fig). Horse to midge transmission rate did slightly influence the outbreak dynamics, which indicated that despite being unknown, factoring this into the model may not result in significantly different overall results. Midge control, resulting in a higher midge mortality rate or reducing the ability for midges to bite horses, impacted the outbreak dynamics, but less than altering vaccination parameters. Finally, changing the infectious period of surviving horses through enforcing euthanasia of infected horses only resulted in smaller outbreaks if implemented very quickly. Otherwise, this was deemed to be an ineffective route for controlling AHS.

## Discussion

This project aimed to explore when and which regions of the South African AHS control zone were most likely to demonstrate an outbreak if a latently infected horse were to be imported. The results of the simulation presented in this paper demonstrated the African horse sickness outbreak dynamics within the control area in South Africa considering the current known details about the vector population, horse population, and viral transmission in this geographical region. The model was developed with the most recently determined input parameters by Fairbanks *et al*. (2022) [14], and despite great heterogeneity over the study area and complex transmission dynamics, the model resembled infection biology as closely as possible while attempting to avoid overcomplication of the model.

Output parameters included the total number of horses affected, number of horses infected at the peak of the outbreak, length of the outbreak, time to the peak of the outbreak, R_v_, and re-emergence. It was important to consider all these concurrently to identify which areas are most at risk for a severe outbreak in the control area. A more severe outbreak was considered to be one which affected higher numbers of horses. However, considering this alongside the outbreak length, time to peak infections, and how many horses were infected at the peak was important because a slower outbreak with a smaller number of infected horses at the peak allowed more time for control measures to be implemented to mitigate further spread. This would mean the potential outbreak size of the simulation was less likely to be achieved in reality.

From the maps in Figs 5 and S3A–S3E, the surveillance and free zone cells demonstrated outbreaks where more horses were infected both at the peak and overall compared to the protection zone. This was not surprising since the vaccination cover in these areas was set at 50% and there was a higher horse population, whereas the protection zone had 95% coverage and a smaller horse population. Outbreak length and time to peak infection appeared to follow a similar trend geographically to horse numbers, but less dramatically. In previous outbreaks up to 2011, outbreaks were managed by increased vaccination efforts, movement control, active surveillance, and increased education. However, in 2014, 2016, and 2021, vaccination was no longer used as a control measure, but the other controls remained in place. The sensitivity analysis showed that by increasing the number of vaccinated horses, the outbreak length and time to peak infection increased to a threshold at around 95% cover and then dramatically decreased when almost no horses were further being infected, thereby initially slowing the outbreak, and providing more opportunity for further controls and testing to be implemented. Increasing the vaccination coverage also decreased the number of horses infected at the peak and the total number of horses affected in the outbreak. Nevertheless, vaccination as a control measure, instead of a preventative measure, is often seen as controversial, as not only does it take up to four weeks for an immune response to mount [22] but vaccinating in the warmer months also presents a risk of a further outbreak due to higher midge densities and temperatures, and therefore more ideal environmental conditions for an outbreak to develop if the vaccination strain reverted to virulence.

Not only does vaccination as a control measure potentially cause problems, but vaccination as a preventative measure has also been demonstrated as a risk factor for outbreaks, due to five out of eight outbreaks in the control area since 1999 being attributed to vaccinations [19]. Therefore, vaccination should be used cautiously, and only during suitable months. Currently, the window in which vaccination is allowed is between 1st June and 31st October, however, the results of this research suggest shortening the window to between 1st July and 31st September would be beneficial to reduce the risk of an outbreak both immediately in the north of the surveillance zone and due to overwintering and re-emergence a few months later in the north of the protection zone as well as the north of the surveillance zone. Nevertheless, re-emergence has never been observed in the control area, and therefore it is unknown whether this is a factor which needs to be considered, but a mechanism for this to be a potential risk has been recognised in previous literature [11]. It is possible that the low numbers of midges and weather extremes could lead to stochastic extinction of vectors in certain areas, resulting in a lower probability of overwintering than the model identified. However, the sensitivity analysis in S4 Fig indicated that midge mortality, which is temperature driven, had less of an impact on outbreak dynamics compared to the features of vaccination and biting rate of the midges. Nevertheless, during the months of July to September, in many areas, outbreaks never established and died off with only the imported horse infected, and the few outbreaks which did emerge were slow and affected very few horses. Therefore, these months appeared to be the times at which there was the least risk of an outbreak if one was to be initiated with a vaccination.

The sensitivity analysis assumed that horse movement was not permitted, and therefore also explored vector control and euthanasia as other control options. While euthanasia was not indicated as effective unless implemented very early on, vector control resulting in reduced biting rate and midge population was shown to be effective for controlling an outbreak. This could be achieved through stabling horses at dawn and dusk, as midges are crepuscular in activity. If stabling is not an option, rugging with a Sweet Itch rug (or other rugs impermeable to midges) would also decrease the biting rate. In addition to this, insecticides could increase the midge mortality rate and therefore reduce the local population. Previous research has also suggested that co-grazing with other animals might result in a decrease in the biting rate through a dilution effect [23], however conversely this could potentially result in an amplification of the epidemic if they preferred horses to livestock, or the midge population increased due to a higher availability of suitable breeding sites such as manure [4]. This is particularly relevant for *C*. *bolitinos*, which preferentially uses domestic cattle, buffalo, and wildebeest dung as breeding sites [24], and therefore co-grazing horses with cattle is likely to amplify AHS transmission. While the biting preferences of *C*. *imicola* and *C*. *bolitinos* still require further research, some preliminary studies have shown that they may demonstrate a preference for horses [25,26]. Nevertheless, recent research has identified that *C*. *imicola* has a wide range of potential bloodmeal hosts, including domestic livestock, wildlife, and humans [27].

The basic reproduction number, R_0_, can be used to estimate how many susceptible horses may become infected from one contagious individual at the start of the outbreak. Likewise, R_v_ incorporates a level of immunity through vaccination from the beginning. R_0_ and R_v_ therefore can sometimes also be used to predict the final epidemic size and severity from early in the outbreak. Nevertheless, this is best in a homogenous and non-spatial model where there are no complexities in terms of heterogenous populations and spatial relationships [28]. In these cases, the epidemic likelihood is better measured by the reproduction ratio of the index farm (R_i_) and the impact of the epidemic can be predicted by calculating the average of the second-generation reproduction ratio (R_i_^(2)^) in a defined area around the index farm. In other words, R_0_ (or R_v_) is best used to determine the potential initial spread of an infection in a population, rather than determine the severity of the outbreak in a heterogenous model like African horse sickness. This was clearly demonstrated in the maps in S3E Fig; winter months, which had large areas where R_v_ was under 1, often demonstrated a lack of ability for an outbreak to establish, but the warmer months, where R_v_ varied considerably, R_v_ was not higher in the areas where the total number of affected horses were higher, nor lower where a lower number of horses were affected. Therefore, for this model, R_v_ should not be interpreted as an indicator of severity of the outbreak, but rather the outbreak potential if the model was more homogenous.

**Fig 7:**
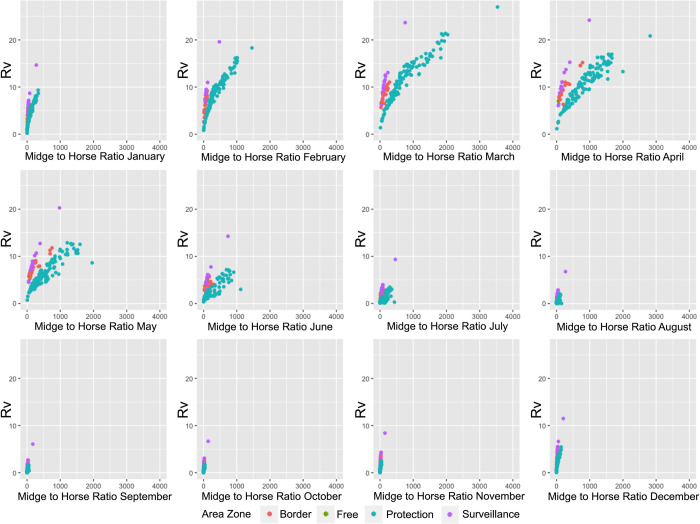
Graphs showing the relationship between the reproduction number (R_v_) and the midge to horse ratio. Scatterplots for each simulation start month, demonstrating how the midge:horse ratio and R_v_ vary depending on the AHS control zone.

In agreement with previous AHS models and demonstrated in the graph in Fig 7, R_v_ is strongly correlated with the vector to horse ratio in the warmer months in all three zones, particularly the protection zone [5,28]. However, the higher ratio might have been influenced by livestock density in the area, and therefore the number of vectors to feeding hosts could have been lower than calculated. Unfortunately, this information is currently unknown. In addition to this, many additional parameters such as temperature, horse demographics, horse and midge populations of neighbouring cells, and vaccination status, contribute to the establishment of an epidemic and there was significant heterogeneity between cells in our model. Therefore, it is important to carefully interpret R_v_ maps as showing potential risk, rather than providing any information about outbreak dynamics.

The model presented in this paper factored in several new elements while building on previous models [5,13,14,28] and gave considerable insight into the dynamics of African horse sickness outbreaks in the control area. However, there are still some areas which could be improved. For example, this model assumed that the susceptible population of horses had no previous immunity. Nevertheless, there have been outbreaks of AHS in the control area in the past, as well as the presence of horses which have been imported from endemic areas, and therefore some non-vaccinated horses may already have a natural immunity to specific serotypes of the virus if they have survived an outbreak in the past. There is known cross-protection between serotypes 1, 5, and 9 by serotypes 2, 8 and 6 respectively [29], so even if a previous outbreak was not of the same serotype, there may be some effective underlying immunity. In addition to this, susceptible horses which had been vaccinated in previous years, but not recently, may still have waning immunity. Therefore, a small protection factor could have been factored into the model to account for a potential underlying natural immunity in some susceptible horses, or an additional compartment of horses could be added to the model for horses with partial immunity.

Another way in which the model could have been improved was with regard to the midge population assumptions. Some areas of the geographical region studied had very few or no midge trapping data entries, and therefore an interpolation map was used to estimate the midge population. The data extracted from the interpolation map was then used to fit a Periodic Gaussian function, which in some cells fit better than others (S1B Fig). The model could have been better informed if a combination of raw data and machine learning techniques were used to estimate midge populations. There have been many studies to determine methods of producing predictive midge abundance maps and the risk for vector-borne diseases. Many environmental factors play a role, and a perfect model is difficult to develop due to the complex relationships between the numerous environmental variables influencing midge population density [30]. However, land surface temperature (LST), in particular minimum LST, and normalised difference vegetation index (NDVI) from satellite imagery have been used to develop prediction models of *C*. *imicola* abundance relatively successfully [31]. Additionally, geographic information systems (GIS) and artificial neural networks (ANN) have been used to investigate the relationships between midge abundance and temperature, rainfall, humidity, altitude, proximity to clay and water bodies, NDVI, LST and livestock density [30], all of which appear significant in the predictive accuracy of *C*. *imicola* and *C*. *bolitinos* abundance. The most recent 2021 outbreak in Cederberg was an example of how the environment plays a role in the transmission of AHS, since it is currently believed that wind dispersal of infected midges may have resulted in the outbreak occurring, and the mountainous terrain in the west and south contributed to limiting the spread of the outbreak [20]. Therefore, the model could be improved to factor in environmental variables which may contribute to the initial risk or spread of the outbreak through influencing midge dynamics, particularly where the land has been utilised for agricultural practices or residential development, rather than left in its natural state. This may allow for it to better inform veterinary officials that perform surveillance or control measures.

Finally, this model used AHS parameters which were determined by an extensive literature review which explored all nine natural AHS serotypes. However, historical outbreaks in the control area have not always been attributed to natural infections. Five out of eight recorded outbreaks since 1999 have been induced through reversion to virulence or reassortment of vaccine strains, and it is currently unknown whether transmission of infections sourced from a vaccination follows the same transmission biology as natural infections. Further research into the transmission dynamics of vaccine-associated outbreaks would enable the model to be better informed to ensure that the simulations in this area account for all potential inciting phenomena. Nevertheless, with the assumption that vaccine-associated infections mimic natural infections, this model still demonstrates how outbreak dynamics may differ across the geographical region and provides insight into areas which may demonstrate more severe outbreaks than others.

In conclusion, the model presented in this study has allowed for further research into AHS outbreaks in the South African control zone, through enhancing previous models by incorporating vaccination and midge movement potential. The model has shown that there are geographical differences in outbreak initiation and dynamics across the control area and highlighted that more severe outbreaks are likely to occur in the warmer months and in the surveillance or free zones. However, the sensitivity analysis indicated that control measures such as vaccination and vector control are potentially effective to manage the spread of an outbreak. In addition to this, shortening the vaccination window to July to September may be beneficial to reduce the potential occurrence of vaccine-associated outbreaks, since these have been shown to be a problem in this area in the past.

## Methods

### Data collection and preparation

To develop the model, data were required to understand horse population size, horse age, vaccination coverage, midge density, and temperature. The following sources were used to collect the required data:

A base map vector layer of the area was provided by SA Equine Health and Protocols (SAEHP) and loaded into QGIS 3.16.3 using the coordinate reference system WGS 84. The base map displayed polygons of municipalities, labelled with their relevant zones of the AHS control area. A 15 by 16 cell grid was overlayed on the base map, whereby each cell measured 0.21 degrees in length and width, equating to approximately 450 ellipsoidal sq.km. In this grid, 153 cells covered the land mass of the study area and were used in the simulation (Fig 1A).

Historical satellite temperatures recorded from 1970 to 2020 were downloaded from WorldClim as a raster layer at approximately 1km resolution (30 seconds) [32]. Mean temperature was extracted for all overlaid grid cells for each month of the year. A sine wave function was fitted to the mean monthly temperature data throughout the year for each cell using the least sum of squares (S1A Fig).

The horse population raster layer was developed for an exposure risk assessment of AHS in the control area through the legal movement of horses by Grewar *et al*. (2021b) [33]. This was formulated using a global distribution raster of population data based on 2002 census figures and provided as a raster layer with pixels measuring 0.0833 decimal degrees on each edge. The mean horse population was extracted for each overlaid grid cell from the raster. Horses represented the largest sized host population, and are spatially separate from others, such as zebra. Therefore this study followed the convention of modelling horses alone, since they are the dominant hosts.

Horse vaccination coverage data for each zone were provided by SAEHP, which publishes vaccination permission reports for the controlled area annually [34]. While vaccination coverage is known, vaccination efficacy research is limited, and field outbreaks demonstrate that not every vaccinated horse has developed an appropriate level of immunity to prevent infection. While vaccine efficacy may also vary among serotypes, they were not considered individually because the model was developed using parameters which were informed by a systematic review exploring all nine serotypes [14]. Molini *et al*. (2015) demonstrated that the immune response to vaccination followed a logistic growth curve. Specifically, the number of seropositive horses and serum neutralizing titres were variable until eight vaccinations (corresponding to 6 years old) [10]. To account for a heterogenous population between grid cells in terms of age of horses and prior vaccination, a parameter which we refer to as the ‘vaccination protection factor’ was applied to represent how likely a horse is to be protected as a result of their vaccination. This equated to an estimation of vaccine efficacy for the horse population. To determine the vaccination protection factor, the proportion of seropositive horses for each age and serotype in Molini’s paper were taken to represent vaccine efficacy for that serotype. This estimate was then reduced by 5% to account for individual horse variation in response to vaccination, for example, horses which cannot mount a full immune response due to age, co-morbidity, or failure in vaccine efficacy such as incorrectly stored vaccines or breaks in the cold chain (O Koekemoer 2021, Research Specialist Virology at OBP, personal communication, 6 May 2021). These figures were then multiplied by the proportion of horses in each age category of the population, in each zone of the control area, based on a gamma distribution of ages produced from the mean age and standard deviation of the known horse population in the control area (S2 Fig). The results were then totalled to give a protection factor for each serotype and averaged to provide an overall age-dependent vaccine protection factor for each zone. The relevant protection factor was then assigned to each cell based on the zone in which it resided in. For cells that spanned the border of two zones, the mean of the two protection factors was assigned to that cell.

Midge population data were provided by the Agricultural Research Council and compiled by Labuschagne in 2015 as part of her Doctor of Philosophy (Entomology) [35]. *Culicoides spp*. midges were collected using 220v UV light traps from surveys over a period of 20 years. The midges were then separated from other insects and identified to species level. Only trap counts of *Culicoides imicola* Kieffer and *Culicoides bolitinos* Meiswinkel were used for this research, as they are the local competent vectors for AHS [24]. For each month of the year, an inverse distance weighted interpolation map was developed using the default power of 2 based on point data containing trap counts. To represent the seasonal variation in midge counts, a Periodic Gaussian function was fitted to the average midge number, y, for each cell and each month of the year using the least sum of squares (S1B Fig):


$$y=\delta \;\exp\left(-s({cos}^{2}(\pi \frac{t}{\sigma -\varphi })\right)$$

where *t* is the time along the x-axis, and the parameters *δ*, *s*, *σ*, and *φ* were fitted where *δ* influenced the height of the peaks, *s* influenced the depth of the troughs, *σ* influenced the frequency of the wave, and *φ* influenced the position on the x-axis related to *t*.

The model and data analysis were performed using R (R Core Team 2020) and QGIS 3.16.3 (QGIS.org, 2021, QGIS Geographic Information System. QGIS Association. http://www.qgis.org).

### Model description

The vector-host model used for this research was a deterministic compartmental model, similar to those of Backer and Nodelijk (2011) [4] and Fairbanks *et al*. (2022) [14]. Table 1 demonstrates the parameters used in the model.

The main differences compared to Backer’s model were the addition of a vaccinated horse compartment and a spatial component where midge dispersal is represented by interaction between the central cell of interest and neighbouring cells using a coupling parameter. Fig 1B shows a schematic of the different compartments and their relationships. In the model, a horse was either susceptible (*S*_*H*_), vaccinated (*V*_*H*_), latent (*E*_*H*_), infectious ($I_{H}^{1}$ and $I_{H}^{2}$), recovered (*R*_*H*_) or dead (*D*_*H*_). Dead horses were removed from the first infectious disease compartment based on the defined disease mortality rate (*m*_*H*_) whilst surviving horses moved to the second infectious compartment and recovered. The natural mortality rate of horses was not included, as the length of infection was significantly shorter than the life span of a horse. The latent (*E*_*H*_), first infectious ($I_{H}^{1}$) and second infectious ($I_{H}^{2}$) compartments were split up into stages which allowed the duration of them to follow a gamma distribution, as defined by Fairbanks *et*. *al* (2022) [14].

Midge vectors were either susceptible (*S*_*V*_), latent (*E*_*V*_) or infectious (*I*_*V*_). The latent midge compartment (*E*_*V*_) was also split up into stages. There was no recovered compartment because the life span of the vectors was so short that the vectors remained infectious for their whole lives. The natural mortality of the vectors (*μ*_*V*_) was removed from each vector compartment and vectors were replaced by the birth of susceptible vectors. The number of infectious vectors (*I*_*V*_) was multiplied by the biting rate (*α*) to give the number of infectious bites in a day (*αI*_*V*_). The fraction of susceptible hosts in the total number of the population ($\frac{S_{H}}{N_{H}}$) was then equal to the probability that an infectious bite falls on a susceptible host. This was also the same for vaccinated hosts ($\frac{V_{H}}{N_{H}}$). Nevertheless, a bite did not always mean the virus was successfully transmitted, and therefore the equation was multiplied by the probability of transmission success from a vector to a susceptible host (*p*_*V*_), or vector to vaccinated host (*p*_*V*_(1−*τ*)), where the probability was adjusted by the protection factor (*τ*) of the vaccine as described earlier as being derived from research by Molini *et al*. (2015)[10]. As a result, the transmission rate from the infectious vector to the susceptible or vaccinated host was calculated by *αp*_*V*_*I*_*V*_*S*_*H*_/*N*_*H*_ or *αp*_*v*_(1−*τ*)*I*_*V*_*V*_*H*_/*N*_*H*_.

The transmission of the virus from an infectious host to a susceptible vector followed a similar equation. The number of biting susceptible vectors per day (*αS*_*V*_), the probability that the virus was successfully transmitted from an infectious host *p*_*H*_ and the probability that the midge bit an infectious host ($(I_{H}^{1}+I_{H}^{2})/N_{H}$), were multiplied together to give the equation $\alpha p_{H}S_{V}(I_{H}^{1}+I_{H}^{2})/N_{H}$. In addition to this, the derivative of the fitted Periodic Gaussian functions (Ω), was proportionally added to each midge compartment to resemble seasonal changes in the total midge population.

The vector mortality rate (*μ*_*V*_), extrinsic incubation rate (*υ*), and biting rate (*α*) were all influenced by temperature (*T*), where warmer temperatures shorten the lifespan of the midges, shorten the extrinsic incubation period, and increase the biting rate [11]. As a result, the following equations were derived [4]:


α(T)=0.015T−0.125

υ(T)=0.0085T−0.0821

$$\mu_{V}(T)=0.015\;exp\;(0.063T)$$

Therefore, the horse and vector compartments were derived from the following equations using the parameters in Table 1:


$$\frac{dS_{V}}{dt}=\mu_{V}N_{V}-\alpha p_{H}\frac{S_{V}(I_{H}^{1}+I_{H}^{2})}{N_{H}}-\mu_{V}S_{V}+\Omega \frac{S_{V}}{N_{V}}$$

$$\frac{dE_{V,1}}{dt}=\alpha p_{H}\frac{S_{V}(I_{H}^{1}+I_{H}^{2})}{N_{H}}-(k\upsilon +\mu_{V})E_{V,1}+\Omega \frac{E_{V,1}}{N_{V}}$$

$$\frac{dE_{V,i}}{dt}=k\upsilon E_{V,i-1}-(k\upsilon +\mu_{V})E_{V,i}+\Omega \frac{E_{V,i}}{N_{V}}$$

for2≤i≤k

$$\frac{dI_{V}}{dt}=k\upsilon E_{V,k}-\mu_{V}I_{V}+\Omega \frac{I_{V}}{N_{V}}$$

$$\frac{dS_{H}}{dt}=-\alpha p_{V}\frac{I_{V}S_{H}}{N_{H}}$$

$$\frac{dV_{H}}{dt}=-\alpha p_{V}(1-\tau )\frac{I_{V}V_{H}}{N_{H}}$$

$$\frac{dE_{H,1}}{dt}=\alpha p_{V}\frac{I_{V}S_{H}}{N_{H}}+\alpha p_{V}(1-\tau )\frac{I_{V}V_{H}}{N_{H}}-l\varepsilon E_{H,1}$$

$$\frac{dE_{H,i}}{dt}=l\varepsilon E_{H,i-1}-l\varepsilon E_{H,i}$$

for2≤i≤l

$$\frac{dI_{H,1}^{1}}{dt}=l\varepsilon E_{H,l}-n^{\left(1\right)}\gamma^{\left(1\right)}I_{H,1}^{1}$$

$$\frac{dI_{H,i}^{1}}{dt}=n^{\left(1\right)}\gamma^{\left(1\right)}I_{H,i-1}^{1}-n^{\left(1\right)}\gamma^{\left(1\right)}I_{H,i}^{1}$$

$$for\;2\leq i\leq n^{\left(1\right)}$$

$$\frac{dI_{H,1}^{2}}{dt}=(1-m_{H})n^{\left(1\right)}\gamma^{\left(1\right)}I_{H,n^{\left(1\right)}}^{1}-n^{\left(2\right)}\gamma^{\left(2\right)}I_{H,1}^{2}$$

$$\frac{dI_{H,i}^{2}}{dt}=n^{\left(2\right)}\gamma^{\left(2\right)}I_{H,i-1}^{2}-n^{\left(2\right)}\gamma^{\left(2\right)}I_{H,i}^{2}$$

$$for\;2\leq i\leq n^{\left(2\right)}$$

$$\frac{dR_{H}}{dt}=n^{\left(2\right)}\gamma^{\left(2\right)}I_{H,n^{\left(2\right)}}^{2}$$

$$\frac{dD_{H}}{dt}=m_{H}n^{\left(1\right)}\gamma^{\left(1\right)}I_{H,n^{\left(1\right)}}^{1}$$

To consider the spatial spread of disease, we adopted a metapopulation approach using a grid comprised of 158 cells, of which the shape and size aligned well with several aggregated pixels of the GIS raster layers containing population and environmental data, and large enough to contain at least two horses. Metapopulation models have been previously used to model livestock diseases [37,38], with research showing that grid-based metapopulation models can provide accurate predictions of the spread of disease [39]. Within a cell, it was assumed that the population was well mixed, whilst between cells, a coupling model was utilised to capture the risk of transmission between cells. This way, infected midges could cross the border of their original cell to a neighbouring cell to bite a susceptible or vaccinated horse, and likewise susceptible midges could cross the border to bite an infected horse. Movement of horses was not included as it was considered that all horse movement would cease following a positive case. The force of infection (*ρ*) between the cells was calculated using a distance (*d*) dependent Gaussian transmission kernel, based on the equation outlined below, where (*κ*) was the kernel parameter which best fits midge transmission during the bluetongue virus outbreaks in Europe in the early 2000’s [36]. This was chosen as bluetongue virus and African horse sickness virus are very similar orbiviruses which share the same vector species and transmission pathways.


$$\rho (d)=\frac{\kappa }{\surd \pi }exp\;(-\kappa^{2}d^{2})$$

Each simulation cell had four neighbours if it was landlocked, with diagonal cells being disregarded to prevent overcomplexity of the model, as it was expected that the risk from cells with shared borders would be significantly higher than diagonal cells. The distance from the central simulation cell (for which the simulation was focussed on) was set at its centroid to the centroid of its neighbours (20 km), whereas the distance from the neighbour centroid to the neighbour centroid was set at 33.3km. This was the average distance between cells which were the neighbours of the simulation cell. This transmission kernel was then multiplied for each cell interaction, by the transmission rates in the susceptible midge, susceptible horses, and vaccinated horse compartments. This output allowed for a continuous kernel to be used in a discrete space, such as a grid cell. The metapopulation model did not expand further spatially into a large network across the study area for two reasons; firstly, to limit overcomplexity and computational time of the model, and secondly due to the limited ability for midges to fly long distances.

The model was simulated for each cell and month (defining a month as 30 days) for 360 days. The simulation was stopped if the latent horses, infected horses, latent midges, and infected midges all dropped under one after a period of 24 days, to prevent the outbreak resurfacing when the temperature increased when realistically it would have died out. The 24-day cut-off point was chosen because it is the sum of the average midge extrinsic incubation period, horse incubation period, and surviving horse infectious period. If a new infection had not developed within that timeframe, the outbreak would not naturally take off again. Outbreak length, time to peak infections, number of infected horses at the peak, number of horses overall affected (recovered or dead), and re-emergence were recorded. Outbreak length was defined as the length of time between initiating the simulation to when the outbreak died out, or the day on which there was less than one infected horse and latent horse after the peak of the initial outbreak. Re-emergence of the virus at a later date could occur if enough infected or latent vectors survived the colder months, which was defined by Boolean expression to indicate whether there was another peak of infectious horses before the simulation time of 360 days came to an end. The time to peak infections was defined as the day on which there was the maximum number of infected horses before the end of the outbreak (or in the cases where re-emergence occurred, before the end of the first wave of the outbreak). The number of infected horses at the peak corresponded with the day on which the outbreak peaked, and the total number of horses affected was calculated from the numbers of dead and recovered horses in the outbreak or first wave in the case of re-emergence.

### Reproduction number

The basic reproduction number R0 signifies the average number of infections an infectious individual can potentially cause during the entirety of its infectious period in an entirely susceptible population. If R0 > 1, the infection has the potential to cause an epidemic, whereas if R0 <1, the outbreak is likely to die out. R0 can be defined using the following equation from Backer and Nodelijk’s model (2011) [4]:


$$R_{0}(T)=\sqrt{\alpha^{2}p_{H}p_{V}\frac{N_{v}}{N_{H}}(m_{H}\frac{1}{\gamma^{\left(1\right)}}+(1-m_{H})(\frac{1}{\gamma^{\left(2\right)}}+\frac{1}{\gamma^{\left(1\right)}})){\left(\frac{k\upsilon }{k\upsilon +\mu_{V}}\right)}^{k}\frac{1}{\mu_{V}}}$$

Since variables such as midge count and temperature change seasonally, the value of R_0_ also changes depending on when and where the outbreak begins. In addition to this, the model in this project included a vaccination protection factor, where

${\alpha p_{V}(1-\tau )(\frac{k\upsilon }{k\upsilon +\mu_{V}})}^{k}\frac{1}{\mu_{V}}$ was the number of vaccinated hosts an infectious vector would infect. This was proportionally combined into the R0 calculation, depending on the number of horses in each compartment which were vaccinated or not. We therefore consider the basic reproduction number in the presence of vaccination, which we refer to as R_v_. This was deemed to be a more appropriate definition than R_0_, as for every simulation vaccinated horses were present at the beginning of the outbreak.


$$R_{v}(T)=\sqrt{(\left(\alpha p_{V}(1-\tau )*\frac{V_{H}}{N_{H}})+(\alpha p_{V}*\frac{S_{H}}{N_{H}}\right))\alpha p_{H}\frac{N_{v}}{N_{H}}(m_{H}\frac{1}{\gamma^{\left(1\right)}}+(1-m_{H})(\frac{1}{\gamma^{\left(2\right)}}+\frac{1}{\gamma^{\left(1\right)}})){\left(\frac{k\upsilon }{k\upsilon +\mu_{V}}\right)}^{k}\frac{1}{\mu_{V}}}$$

### Sensitivity analysis

A sensitivity analysis was undertaken to further explore uncertain parameters relating to vaccination, as well as parameters which may vary if control measures were implemented. Since much is still unknown about AHS vaccines, including knowledge on individual immune response variation (where in this study a 5% reduction from full effect was accounted for to be conservative), the parameters chosen to evaluate included vaccination cover, vaccination protection factors, and transmission from an infected (vaccinated) horse to a vector. In addition to these, parameters which could be influenced by control measures were also analysed. The midge biting rate (*α*) and mortality (*μ*_*V*_) could be altered through vector control such as midge repellents, protective “Sweet Itch” rugs designed to manage horses with *Culicoides* skin sensitivity, insecticides, stabling at dusk and dawn when the vectors are most active, and stabling inside enclosed barns. Moreover, the infectious period of horses who do not naturally die (*γ*^(2)^) could be shortened through elective euthanasia of infected horses.

One hundred equally spaced samples of each parameter were selected and analysed using a “one-at-a-time” (OAT) method. Where there was an unknown range of possible values for a parameter, the samples were selected between an interval of 20% less or more than the value used in the model. However, if a larger range was biologically appropriate (for example, vaccine protection factor and proportion vaccinated could satisfy values between 0–100%) or Fairbanks *et*. *al’s* (2022) systematic review indicated a larger range has been observed, these values were considered [14]. The simulation was run with varying one parameter at a time for cell ID number 84, which was also the cell used as the simulation example. This cell was chosen to demonstrate the simulation and sensitivity analysis as it is in the heart of the study area. It is also in the surveillance zone, so there are similar numbers of vaccinated and unvaccinated horses included, which is important for the sensitivity analysis so that higher levels of vaccination do not mask trends when altering other parameters.

The outcome variables analysed from the sensitivity analysis included the number of horses affected overall, number of horses infected at the peak of the outbreak, days to the peak of the outbreak, length of the outbreak and R_v_ value.

## Supporting information

S1 Fig Sample graphs showing the goodness of fit. (A) Six graphs, of which one is the example simulation cell, and five were randomly chosen, representing the sine waves fitted to the mean temperature data using a sum of least squares method. (B) Similarly, six graphs showing the Periodic Gaussian function fitted to the midge population data.
(DOCX)

S2 Fig Histograms showing the gamma distributions of the horse population ages in each zone of the AHS control area. The mean age of the free zone (3.65 years) was considerably lower than the surveillance (9.56 years) and protection areas (8.01 years). Gamma distributions were calculated using R Studio, using mean and standard deviations of horse population data for registered horses with a date of birth after 1^st^ January 2000, supplied by the SAEHP.
(TIF)

S3 Fig Spatiotemporal maps demonstrating the outbreak dynamics when the simulation was run for 360 days varying the start month between January to December (A-L). (A) Number of days until the peak of the horse infections. (B) Outbreak duration in days. (C) Number of infected horses at the peak of the outbreak. (D) Re-emergence potential. (E) Reproduction number. The base layer of the maps is publicly available to download from the Municipal Demarcation Board of South Africa (2018) [21].
(DOCX)

S4 Fig Line graphs demonstrating the changes in outbreak dynamics using a one-at-a-time sensitivity analysis. The simulation was for the month of March for 360 days when the temperature and midge populations were high, ensuring the outbreak is likely to take off. For each variable (midge mortality, biting rate, vaccine protection factor, horse to midge transmission, infectious period of surviving horses, proportion vaccinated), the simulation was run 100 times, varying the input. Midge mortality and biting rate was recorded as a proportion change, whereby 1.0 was the value used in the main simulation, 0.8 was a 20% reduction in the value, and 1.2 was a 20% increase in the value.
(TIF)

S1 Text Exploration of spatial epidemiology results and real-world data. (DOCX)
