# Correction: Modelling African horse sickness emergence and transmission in the South African control area using a deterministic metapopulation approach

**DOI:** 10.1371/journal.pcbi.1013377

**Published:** 2025-08-12

**Authors:** Joanna N. de Klerk, Erin E. Gorsich, John D. Grewar, Benjamin D. Atkins, Warren S. D. Tennant, Karien Labuschagne, Michael J. Tildesley

## Notice of Republication

This article was republished on July 29, 2025, to correct errors in the data availability statement. Please download this article again to view the correct version. The originally published, uncorrected article and the republished, corrected articles are provided here for reference.

## Supporting information

S1 File Originally published, uncorrected article. (PDF)

S2 File Republished, corrected article. (PDF)
