# Supplementary figures and images for: Modelling African horse sickness emergence and transmission in the South African control area using a deterministic metapopulation approach
Source: PLoS Comput Biol. 2023 Sep 6;19(9):e1011448. doi: 10.1371/journal.pcbi.1011448 (PMC10506717; doi:10.1371/journal.pcbi.1011448)

A
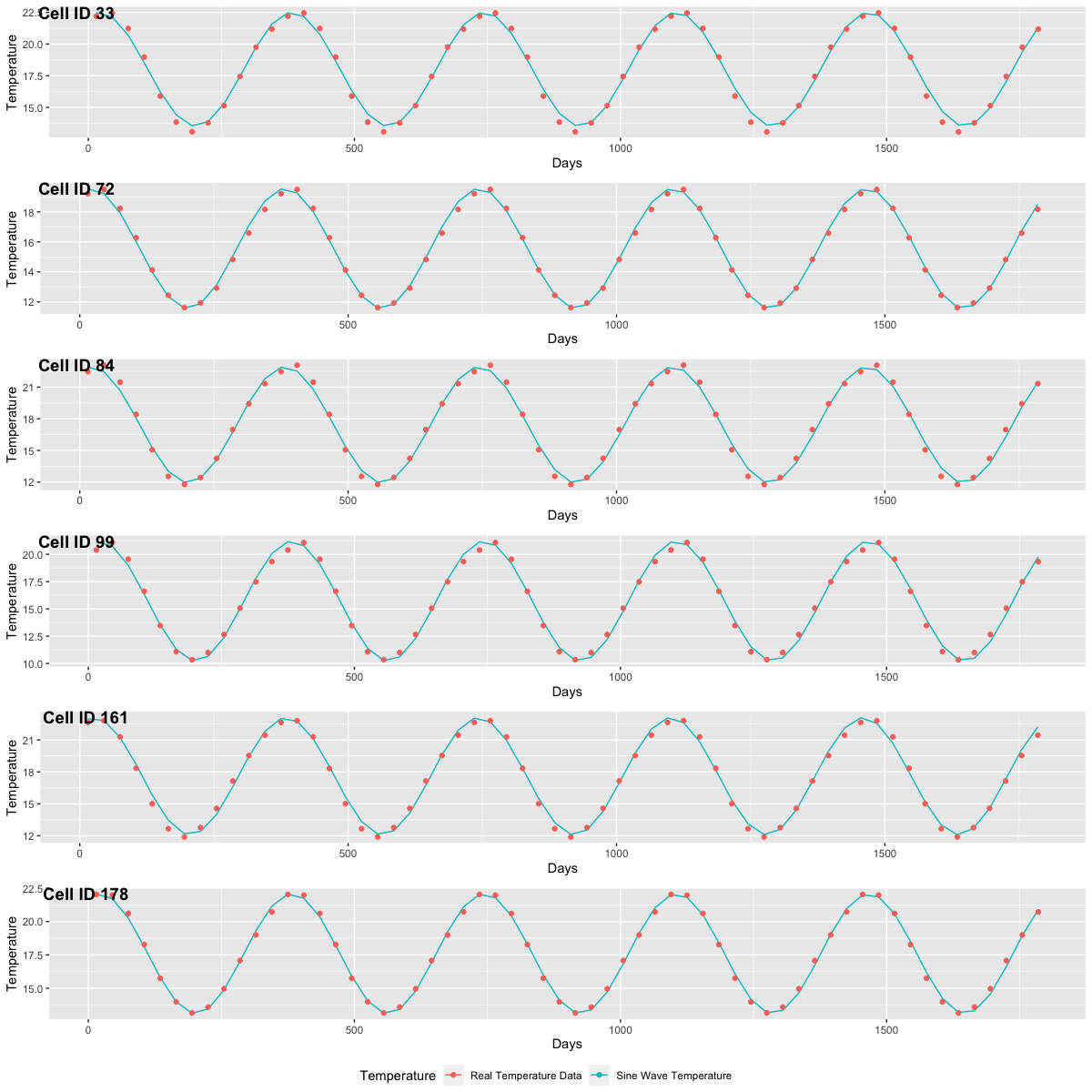
B
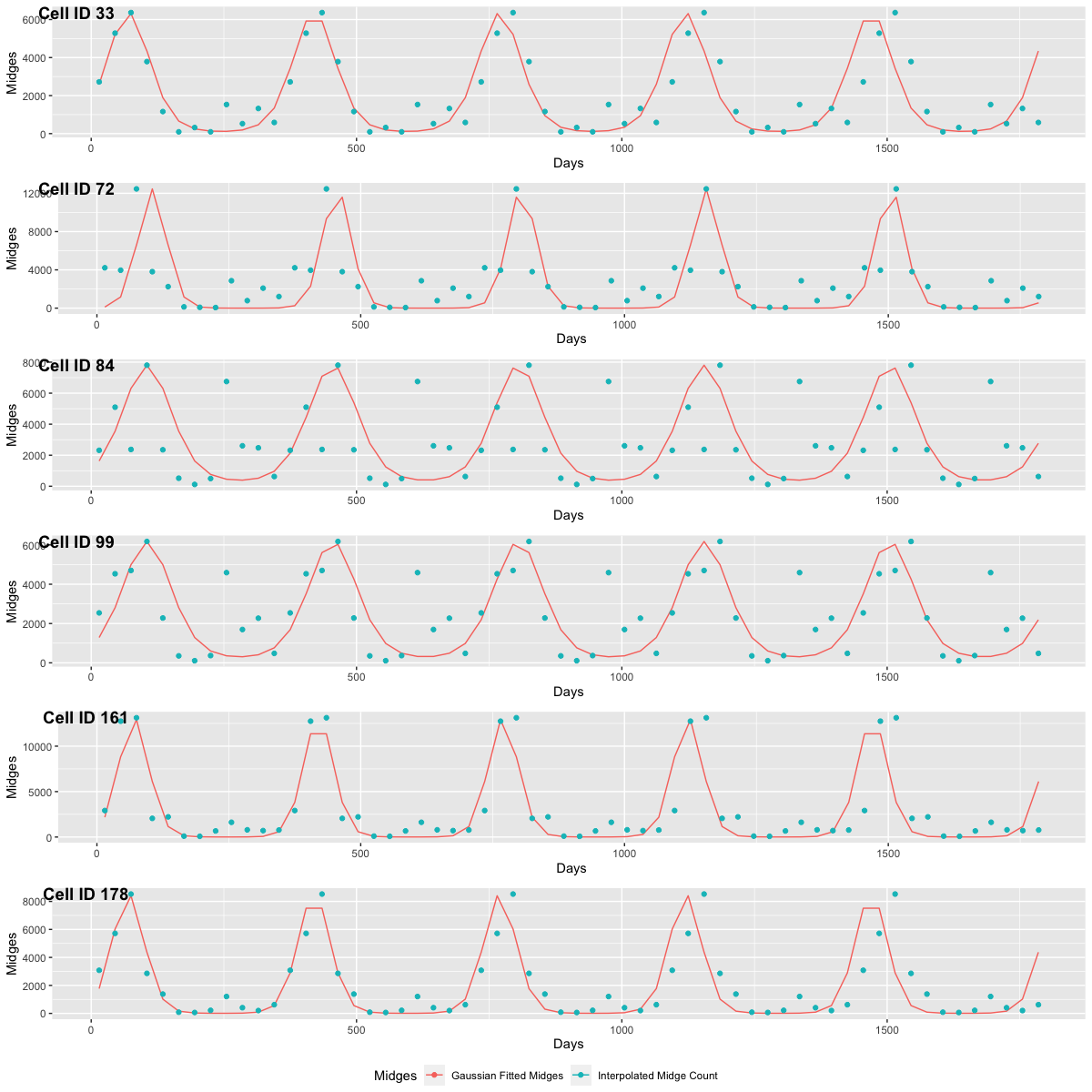

Supplement: S1 Fig — (A) Six graphs, of which one is the example simulation cell, and five were randomly chosen, representing the sine waves fitted to the mean temperature data using a sum of least squares method. (B) Similarly, six graphs showing the Periodic Gaussian function fitted to the midge population data. (DOCX) [file pcbi.1011448.s001.docx]

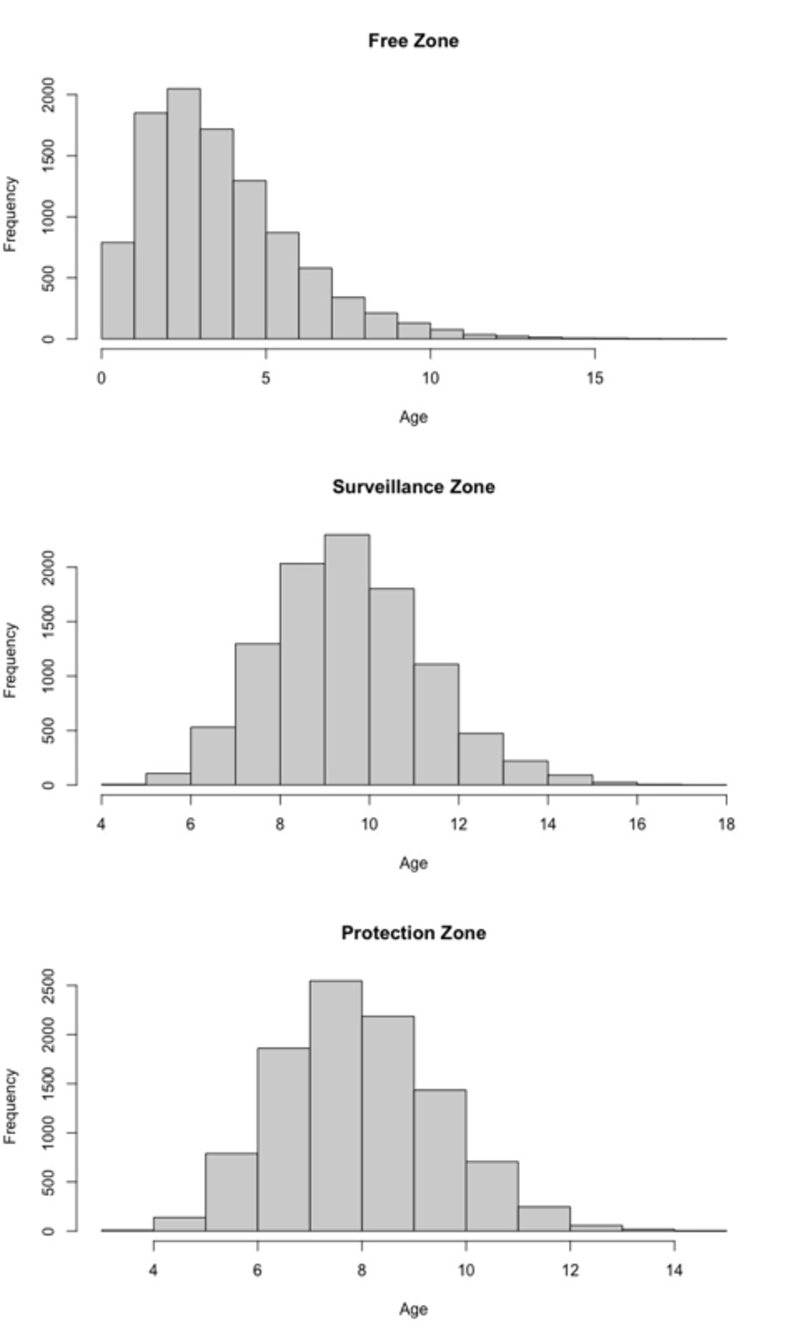

Supplement: S2 Fig — The mean age of the free zone (3.65 years) was considerably lower than the surveillance (9.56 years) and protection areas (8.01 years). Gamma distributions were calculated using R Studio, using mean and standard deviations of horse population data for registered horses with a date of birth after 1st January 2000, supplied by the SAEHP. (TIF) [file pcbi.1011448.s002.tif]

A


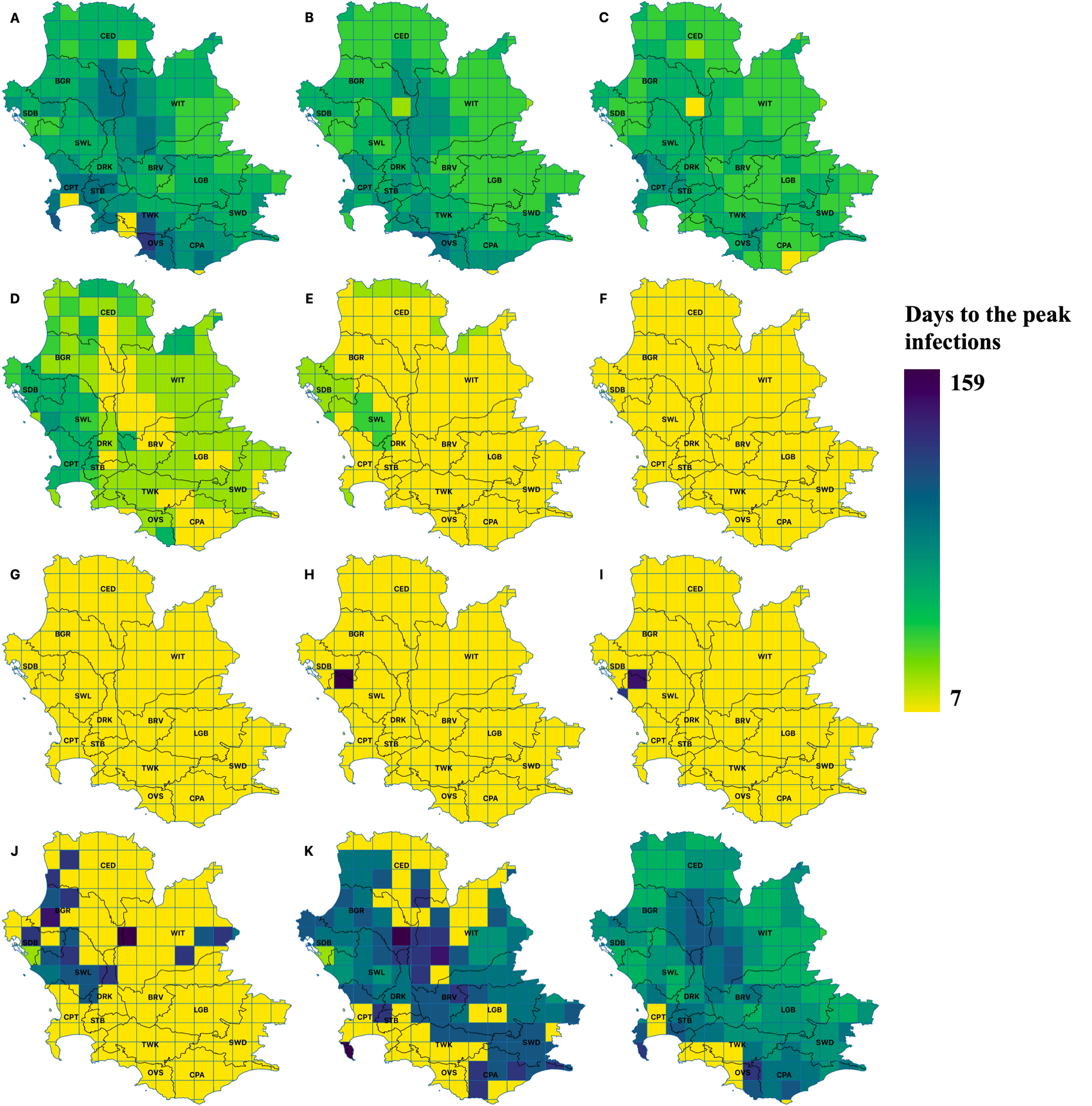


B


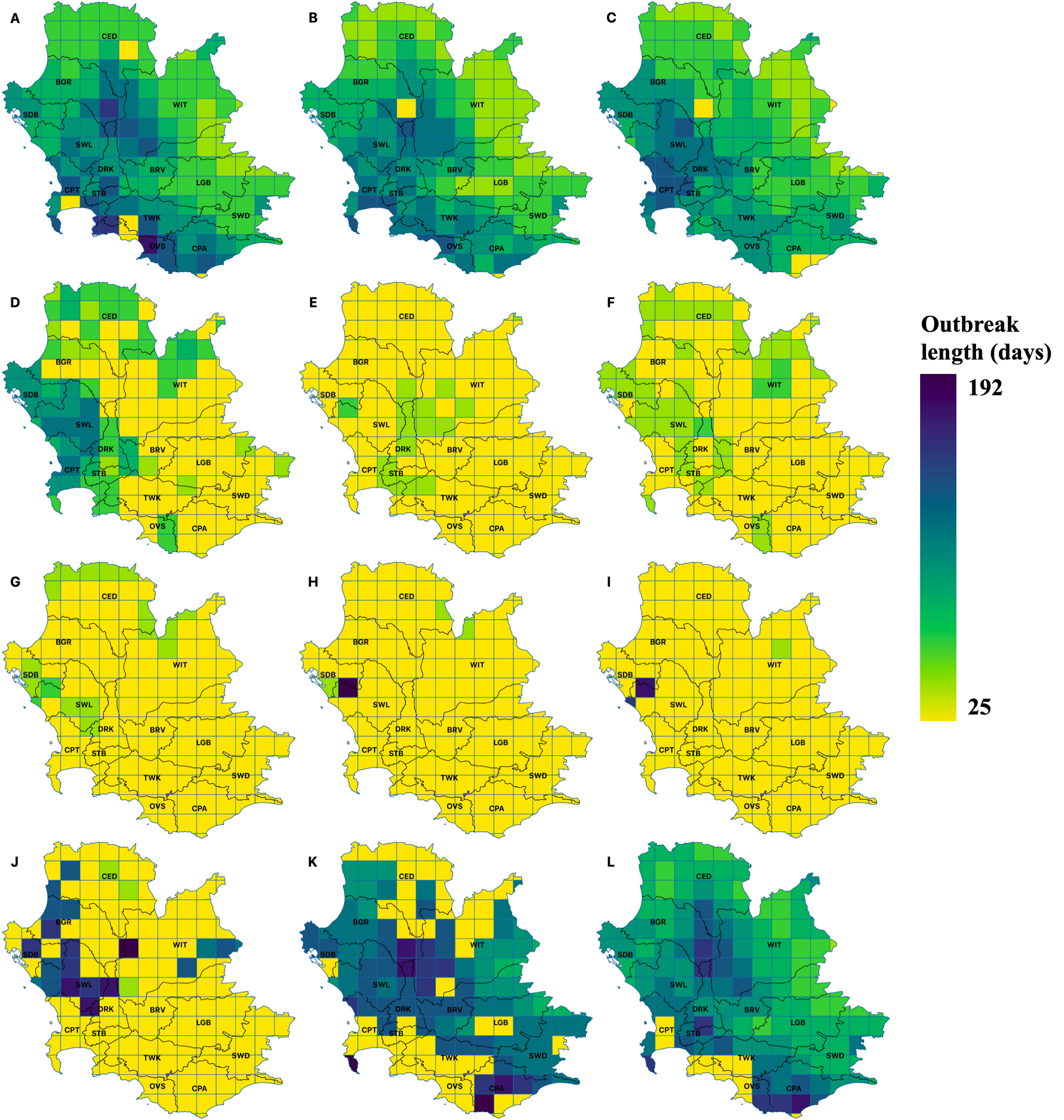


C


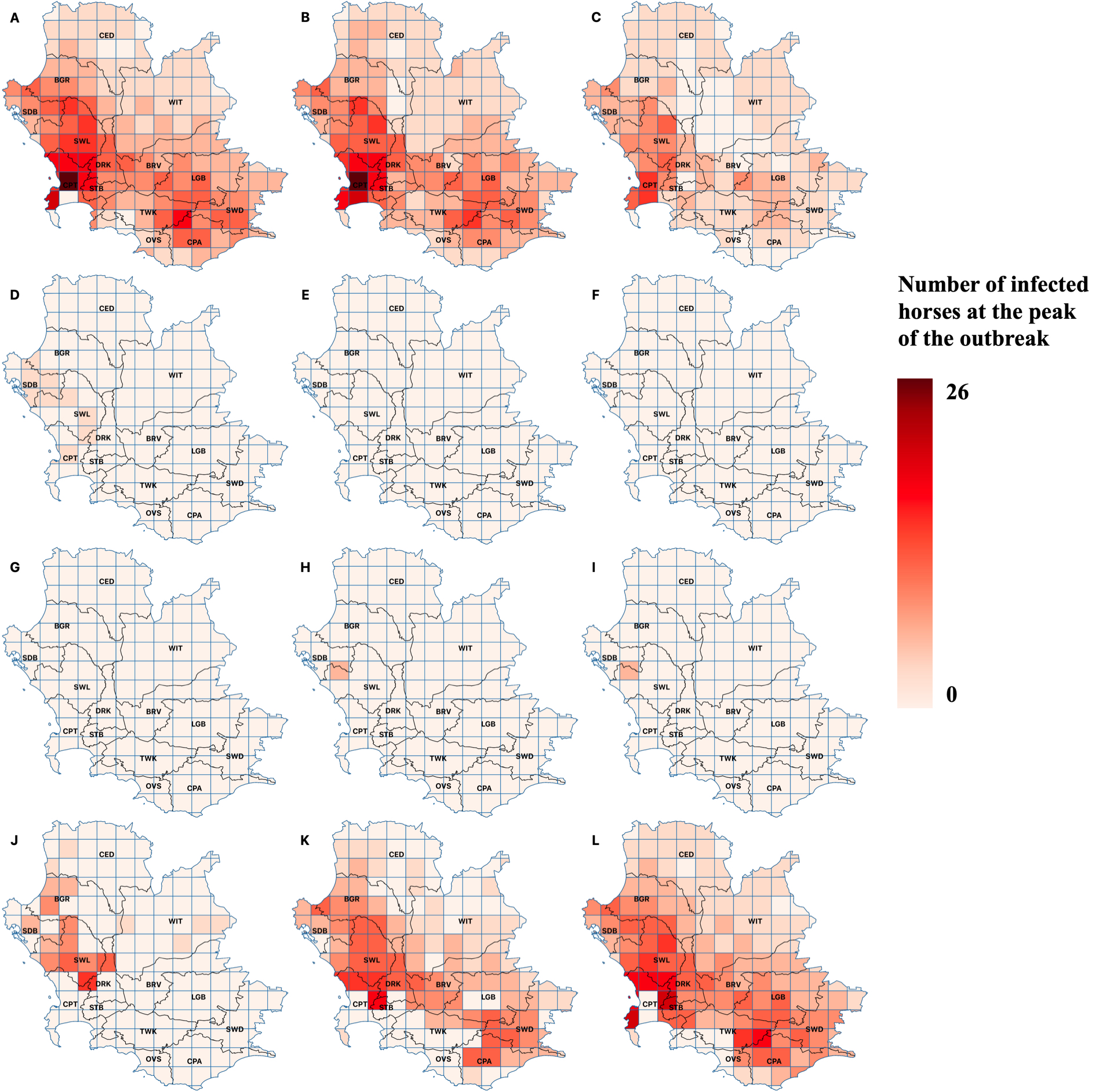


D


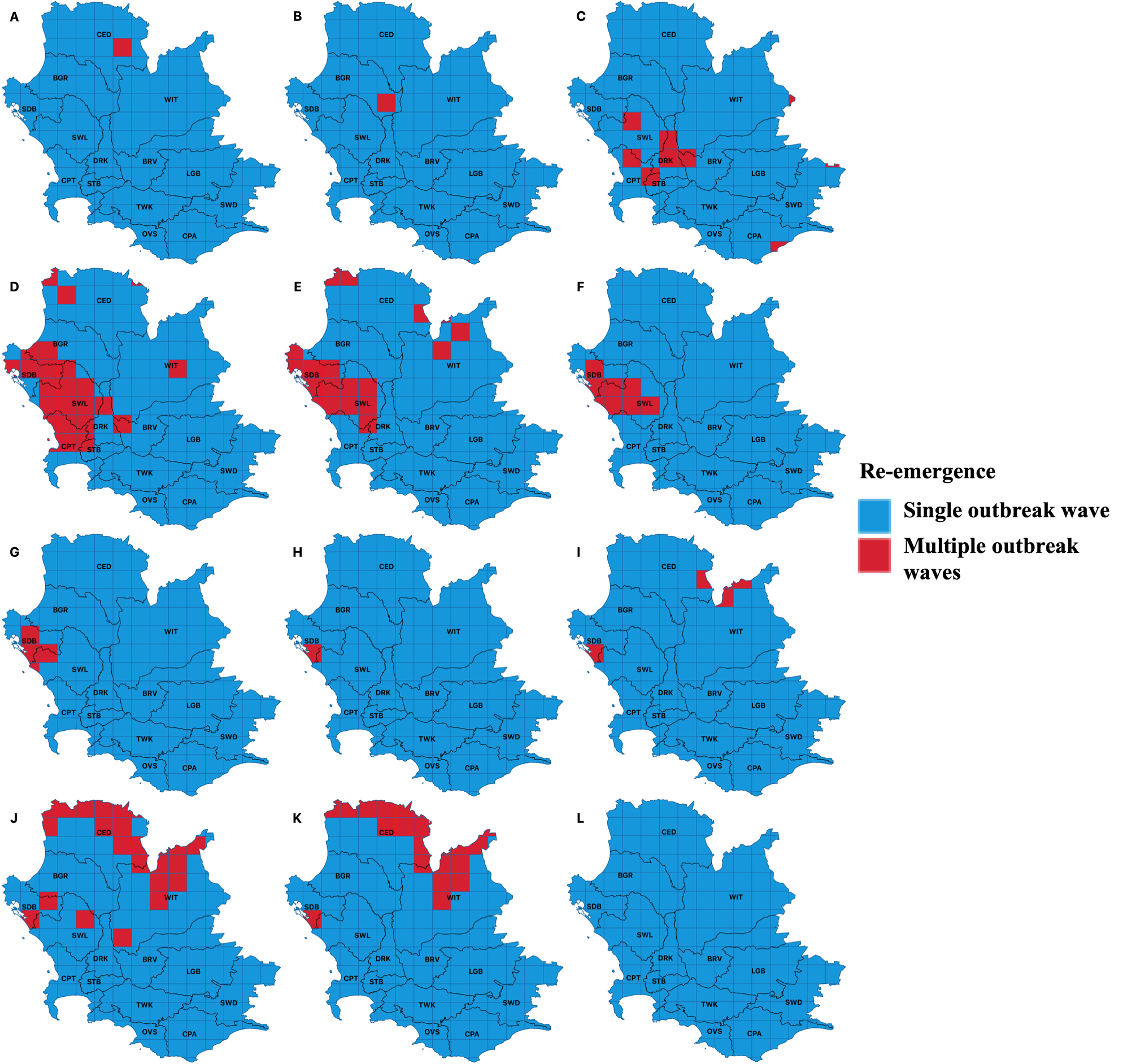


E


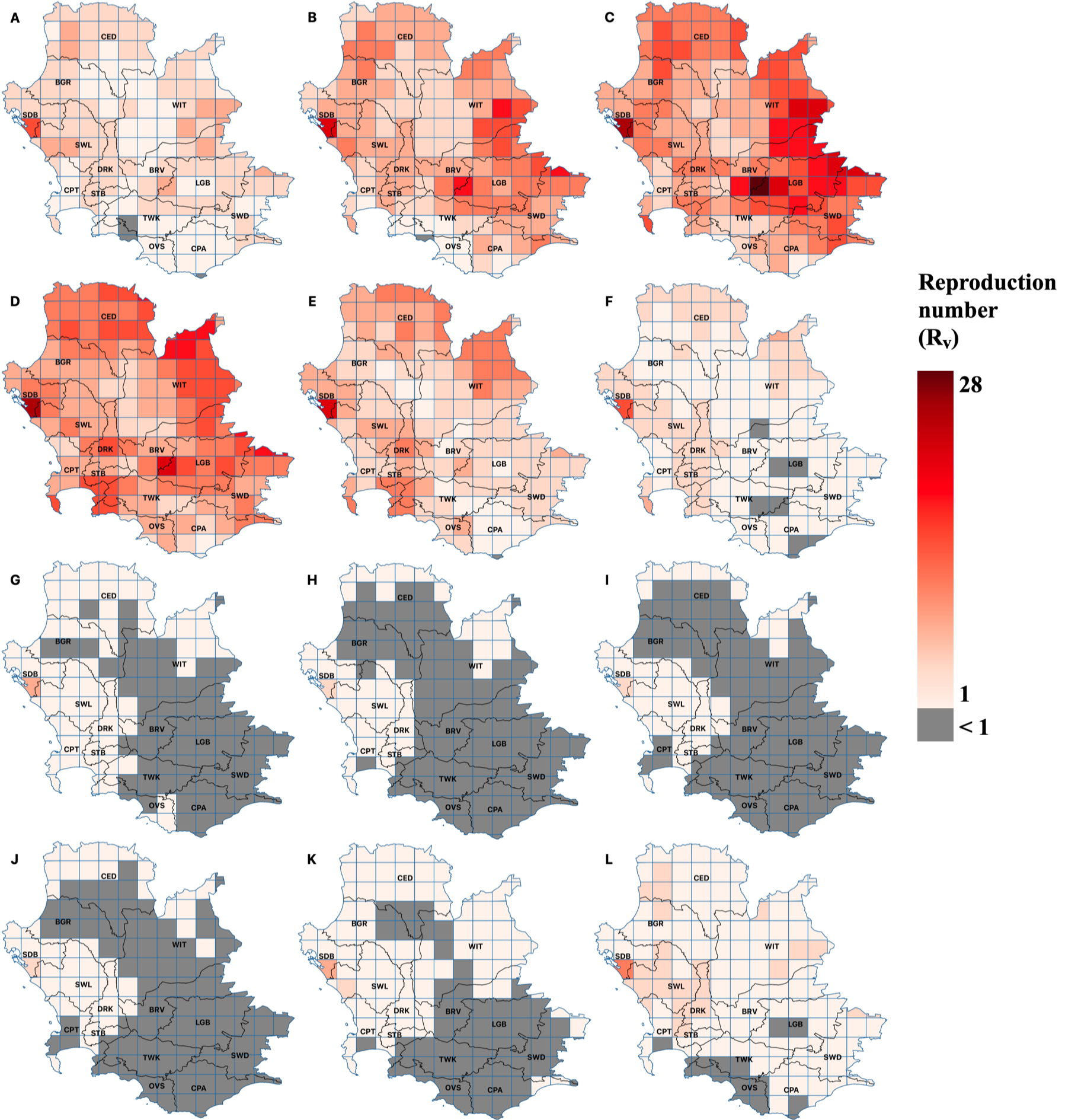

Supplement: S3 Fig — (A) Number of days until the peak of the horse infections. (B) Outbreak duration in days. (C) Number of infected horses at the peak of the outbreak. (D) Re-emergence potential. (E) Reproduction number. The base layer of the maps is publicly available to download from the Municipal Demarcation Board of South Africa (2018) [21]. (DOCX) [file pcbi.1011448.s003.docx]

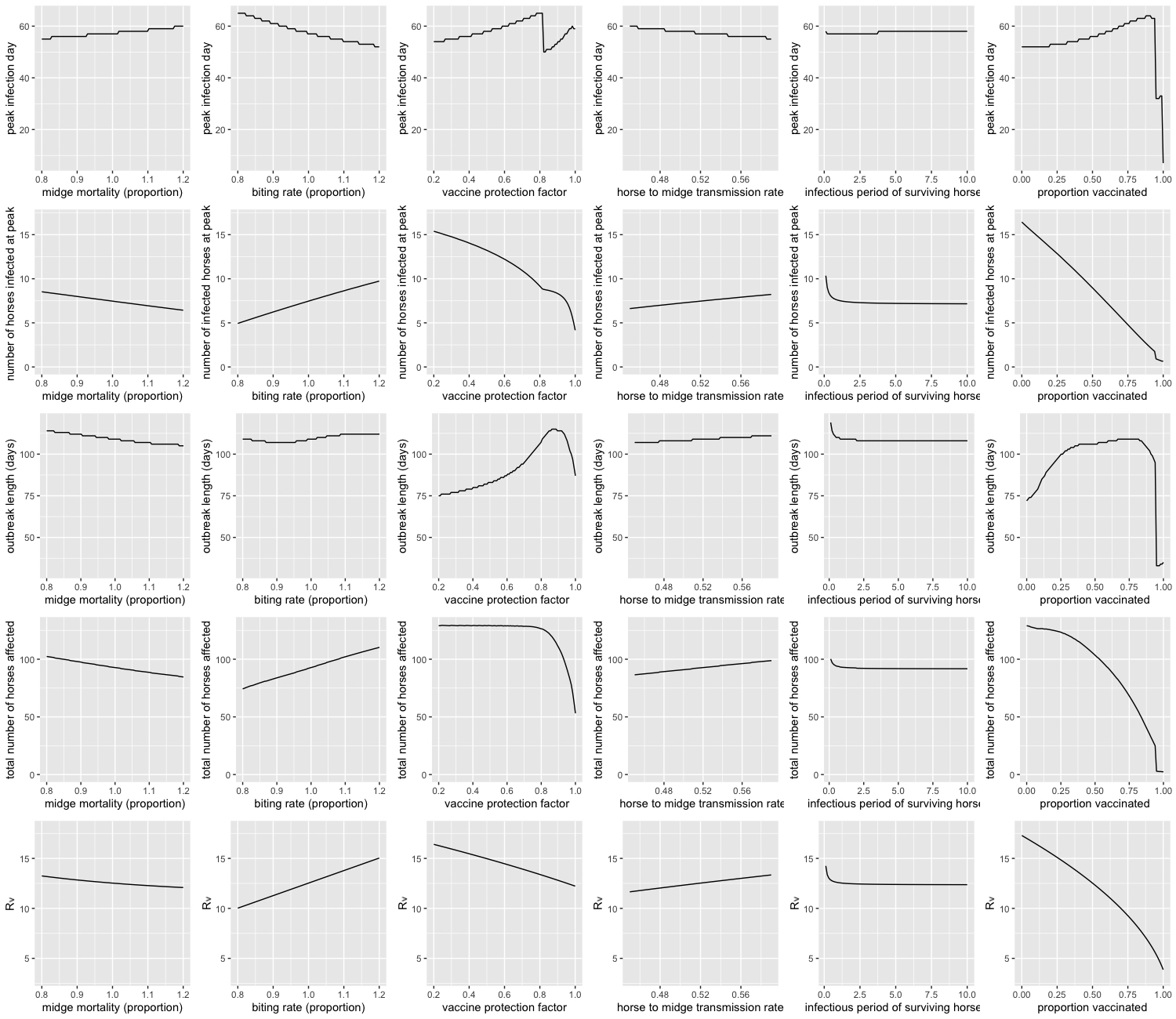

Supplement: S4 Fig — The simulation was for the month of March for 360 days when the temperature and midge populations were high, ensuring the outbreak is likely to take off. For each variable (midge mortality, biting rate, vaccine protection factor, horse to midge transmission, infectious period of surviving horses, proportion vaccinated), the simulation was run 100 times, varying the input. Midge mortality and biting rate was recorded as a proportion change, whereby 1.0 was the value used in the main simulation, 0.8 was a 20% reduction in the value, and 1.2 was a 20% increase in the value. (TIF) [file pcbi.1011448.s004.tif]
