# Supplementary material for: Modelling African horse sickness emergence and transmission in the South African control area using a deterministic metapopulation approach
Source: PLoS Comput Biol. 2023 Sep 6;19(9):e1011448. doi: 10.1371/journal.pcbi.1011448 (PMC10506717; doi:10.1371/journal.pcbi.1011448)
Supplement: S1 Text — (DOCX) [file pcbi.1011448.s005.docx]

# **Supplementary Material 4**

## **Exploration of spatial epidemiology results and real-world data**

In the African Horse Sickness (AHS) control area in South Africa, there have been eight known AHS outbreaks since 1999 for which data have been recorded (Fig 4). They include outbreaks caused by natural imported infection, reversion to virulence of a serotype included in the live-attenuated vaccine (LAV) available in South Africa, and reassortment of genome segments from the LAV polyvalent vaccine (Table 1). The model was parameterised based on data from natural infections, so Table 1 includes model predictions for natural outbreaks only. Specifically, the model explores the potential outbreak dynamics if a latently infected horse were imported into the AHS control area across different locations and different months of introduction.

To visually compare observed outbreak locations with model predicted outbreak dynamics, we plotted outbreak area polygons on the study area map with simulation outputs for the relevant month. This was done by using the most outer case points to encompass all case point data within the polygon. For outbreaks caused by natural infections, simulation outputs were recorded for all grid cells on which the polygons were overlaid, and the mean and standard deviation of those simulations are displayed in Table 1.

Table 1 Comparison of outbreak dynamics between known outbreaks and the model. Mean and standard deviations of all simulation results were recorded for grid cells on which the natural outbreak polygons were overlaid. Data were extracted from SAEHP published material [1, 2] * R_A_  = Reassortment, R_V_ = Reversion to virulence, Natural = wild-type AHS

| Outbreak | Cause* | Outbreak Total Cases | Subclinical Cases | Simulation Cases Mean | Simulation Cases s.d. | Outbreak Length (days) | Simulation Length Mean (days) | Simulation Length s.d (days) |
| --- | --- | --- | --- | --- | --- | --- | --- | --- |
| 1999 Stellenbosch | Natural import | 54 | Unknown | 81 | ± 49 | 57 | 105 | ± 21 |
| 2004 Stellenbosch | R_A_ & R_V_ | 23 | 0 |  |  | 56 |  |  |
| 2006 Robertson | Natural import | 32 | Unknown | 55 | ± 0 | 83 | 66 | ± 5 |
| 2011 Mamre | R_A_ | 84 | 15 |  |  | 66 |  |  |
| 2014 Porterville | R_A_ | 89 | 52 |  |  | 73 |  |  |
| 2014 Robertson | R_A_ | 22 | 17 |  |  | 42 |  |  |
| 2016 Paarl | R_V_ | 21 | 14 |  |  | 32 |  |  |
| 2021 Cederberg | Natural – unknown cause | 37 | 3 | 22 | ± 4 | 46 | 72 | ± 4 |

**Discussion**

A formal validation statistically comparing AHS outbreak duration and size to the model is not possible due to limited information on natural versus vaccine-induced outbreaks, as well as changes in the sensitivity of diagnostic testing over time.

Up until 2014, a vaccination campaign using LAVs was implemented as a control measure [1]. LAVs are the only available AHS vaccine type in South Africa, however, they paradoxically provide a risk for AHS outbreaks. Therefore, from 2014, they ceased to be used as a control measure. Five out of the eight outbreaks recorded have been a result of virulent revertants of the AHS serotype-1 LAV, or reassortants displaying whole genome segments of AHS serotypes -1, -3, and -4 from the LAV available in South Africa [3]. There have been no published studies on whether the transmission dynamics of outbreaks of this nature are similar to wild-type infections, and therefore the infectivity rates, incubation periods and vaccine protection parameters are unknown. Therefore, this method of introduction was not considered. Future research on characterising vaccine-induced outbreaks would be beneficial, to understand whether they share the same infection biology as natural infections. This would allow for more accurate modelling of vaccine-related AHS outbreaks in South Africa.

The model predicts realistic outbreak sizes and durations, but these results need to be interpreted in the context of diagnostic testing and control measures. For the 1999 and the 2006 outbreaks, the simulation over-estimated the number of cases affected, however these outbreaks also included vaccination campaigns, as well as less sensitive diagnostic methods, which combined may have contributed to this statistic. However, in 2021, the simulation output was much closer to the recorded number of cases. The simulation lengths in all cases were no greater than two-fold.

The number of subclinical cases in real outbreaks can be very high (for example, Porterville in 2014 with 58%, Robertson in 2014 with 77%, and Paarl in 2016 with 64% subclinical) and therefore, real-world data may not always detect the true number of cases or duration of the outbreak. The three earliest outbreaks did not record any subclinical cases; however, this is unlikely to be representative of the true situation, and may have been a result of case identification which has improved throughout the years. In 1999, the AHS case definition only included deaths. In 2004, live cases of AHS were identified through viral isolation and serology. Then in 2006, port mortem examination, clinical examination and regular PCR were included to the case definition. After that, in 2011, RT-qPCR became available. And finally, from 2014, sequencing was also available as a diagnostic method [1]. As a result, earlier outbreaks probably missed cases which contributed to the transmission of the virus and therefore the data available is likely to be an underestimation of the true outbreak size, and possibly length.

In conclusion, we believe that the model presented in this research adequately captures the dynamics of AHS outbreaks in the AHS control area. The AHS control area is the only area of South Africa with increased surveillance measures, and therefore data, on the outbreaks for model evaluation. It is important to acknowledge that the data only identifies a proportion of the cases in a limited number of outbreaks, but it still provides a rare opportunity for model evaluation.

**References**

1. Grewar JD. *Outbreaks Of African horse sickness in the Controlled Area of South Africa*. Equine Health Fund, Wits Health Consortium. 2017 [Cited 2022 April 20]. Available from: <https://myhorse.jshiny.com/myhorse/landing/resources/infographics/ahs_outbreaks_ca.pdf>
2. SAEHP. *Information of the AHS outbreak in the Protection Zone of South Africa - Cederberg 2021*. SAEHP. 2021 [Cited 2023 May 29]. Available from: <https://myhorse.jshiny.com/myhorse/2021AHS/outbreak/>
3. Weyer CT, Grewar JD, Burger P, Rossouw E, Lourens C, Joone C, le Grange M, Coetzee P, Venter E, Martin DP, MacLachlan NJ. African horse sickness caused by genome reassortment and reversion to virulence of live, attenuated vaccine viruses, South Africa, 2004–2014. Emerging infectious diseases. 2016 Dec;22(12):2087.
